# In Vitro Study of Multi-Therapeutic Properties of *Thymus bovei* Benth. Essential Oil and Its Main Component for Promoting Their Use in Clinical Practice

**DOI:** 10.3390/jcm7090283

**Published:** 2018-09-15

**Authors:** Sherif T. S. Hassan, Kateřina Berchová-Bímová, Miroslava Šudomová, Milan Malaník, Karel Šmejkal, Kannan R.R. Rengasamy

**Affiliations:** 1 Department of Natural Drugs, Faculty of Pharmacy, University of Veterinary and Pharmaceutical Sciences Brno, Palackého tř. 1946/1, Brno 612 42, Czech Republic; milan.malanik@seznam.cz (M.M.); karel.mejkal@post.cz (K.Š.); 2 Department of Applied Ecology, Faculty of Environmental Sciences, Czech University of Life Sciences Prague, Kamýcká 129, 165 21 Praha 6-Suchdol, Czech Republic; berchova@fzp.czu.cz; 3 Museum of literature in Moravia, Klášter 1, 664 61, Rajhrad, Czech Republic; sudomova@post.cz; 4 REEF Environmental Consultancy Services, #2 Kamaraj Street, S.P. Nagar, Puducherry 605 001, India; cr.ragupathi@gmail.com

**Keywords:** anticancer, antihypertensive, HSV-2, *Thymus bovei* Benth., essential oil, phytochemical profile

## Abstract

*Thymus bovei* Benth. (TB) is an important plant in the traditional medicine of the Mediterranean region. This study investigates the health-promoting properties of TB essential oil (TB-EO) for its possible use in clinical practice with regards to its cytotoxic, anti-herpes simplex virus type 2 (HSV-2), and antihypertensive (through inhibition of human angiotensin-converting enzyme; ACE) properties. The phytochemical profile of EO (99.9%) was analyzed by Gas Chromatography with Flame-Ionization Detection (GC-FID) and Gas Chromatography-Mass Spectrometry (GC-MS). In this study, all biological methods were performed at the level of in vitro studies. The results showed that TB-EO exerted remarked cytotoxic properties against human cervical carcinoma cells, colon cancer cells, and lung adenocarcinoma cells with the half-maximal inhibitory concentration (IC_50_) values of 7.22, 9.30, and 8.62 µg/mL, respectively, in comparison with that of standard anticancer drug cisplatin with IC_50_ values of 4.24, 5.21, and 5.43 µg/mL, respectively. Fascinatingly, TB-EO showed very weak cytotoxicity on the healthy human fetal lung fibroblast cells with an IC_50_ value of 118.34 µg/mL compared with that of cisplatin (IC_50_ = 10.08 µg/mL). TB-EO, its main component geraniol, TB-EO combined with acyclovir (ACV) along with standard ACV, have displayed pronounced inhibitory properties against the replication of HSV-2 with the half-maximal effective concentration (EC_50_) values of 2.13, 1.92, 0.81 and 1.94 µg/mL, respectively, with corresponding selectivity indices (SI) 98.59, 109.38, 259.26 and 108.25, respectively. TB-EO and geraniol at a concentration of 15 µg/mL showed prominent inhibitory activities against ACE with % of inhibition 95.4% and 92.2%, respectively, compared with that of standard inhibitor captopril (99.8%; 15 µg/mL). Molecular docking studies were performed to unveil the mechanism of action of geraniol as well as structural parameters necessary for anti-HSV-2 activity (through the inhibition of HSV-2 protease) and ACE inhibition. This is the first report on the chemical composition of Egyptian TB-EO along with the above-mentioned biological activities. Our results may be considered as novel findings in the course of a search for new and active anticancer, anti-HSV-2 and antihypertensive agents, and expand the medicinal value of this plant and its phytochemicals in clinical practice.

## 1. Introduction

For decades, plants have been known to be a vital source of natural preparations comprising many biologically active molecules that can be used for various applications, such as food additives and health-promoting agents in the form of functional foods and nutraceuticals. Since ancient times, aromatic plants have been widely used as flavorings, and later, they have become a subject for a search of drugs that can be used for the treatment of a wide range of diseases [1,2]. *Thymus bovei* Benth. (*Lamiaceae*), commonly known as “Thyme” is a medicinal and aromatic herb, widely distributed in the eastern Mediterranean regions [3]. This plant is considered to be one of the most important *Thymus* species, due to its high medicinal and curative characteristics. Traditionally, in the North African region, particularly in Egypt, this plant is being used for the treatment of skin and blood infections as well as curing a wide range of microbial infections [4]. It has been stated that this herb has anthelmintic, expectorant, antispasmodic, antiseptic properties due to its content of essential oil (EO) [5]. Moreover, medicinal properties such as curing of injuries, treatment of upper respiratory tract inflammations, and treatment of anorexia, have also been reported [6]. Plant-derived EOs are expected to possess a good bioavailability due to its lipophilic properties, and hence induce curative activities [7].

In recent years, cancer has become the most severe health issue across the globe and is the major cause of mortality worldwide [8]. In this regard, the search for new effective molecules that exhibit antitumor properties against numerous types of cancer has become one of the main topics in the field of natural product research [9]. Plants and their phytochemicals have a rich history of use, and they play a critical role in the development of sophisticated traditional medicine systems, particularly in cancer therapy [10]. EOs derived from various plant species have shown to exert excellent anticancer properties [11,12,13,14]. So far, no reports have demonstrated the anticancer activity of *T. bovei*.

Genital herpes is an incurable viral sexually transmitted disease, induced by herpes simplex virus type 2 (HSV-2). HSV-2 is an enveloped, double-stranded DNA virus of the Herpesviridae family [15]. This virus contaminates the genital mucosa and causes ulcers and lesions in the genital area. Following the principal infection, the virus creates a life-long latent infection within sensory dorsal root ganglia. The occurrence of recurrent infection is mainly generated by reactivation of latent infection, particularly throughout the deficiency of immunity [16,17]. It has been reported that infection with HSV-2 increases the risk of infection with human immunodeficiency virus (HIV), as well as invasive cervical carcinoma. It is known that nucleoside analogs, such as acyclovir (ACV) and its derivatives penciclovir, famciclovir, cidofovir, valacyclovir, trifluridine, and vidarabine are used to treat the HSV-2 infection [18]. However, the development of resistance to antiherpetic medications along with the undesirable side effects claims for the development of new anti-HSV-2 drugs with assorted mechanisms of action [19].

Angiotensin-converting enzyme (ACE) is a zinc-containing metallopeptidase that can elevate blood pressure by converting the inactive decapeptide angiotensin I to the effective vasoconstrictor angiotensin II, in addition, to degrading the hypotensive peptide bradykinin [20]. Due to the fundamental role of ACE in cardiovascular diseases, it has been an attractive target for drug design [21]. Up to date, no investigations have reported the ability of *T. bovei*-derived EO to diminish the risk of hypertension and coronary heart disease by inactivating the enzymatic properties of ACE.

The purpose of this study was to explore the cytotoxic, anti-HSV-2 and antihypertensive activities of *T. bovei* Benth. EO (TB-EO) and its main active compound geraniol, along with characterization of the phytochemical profile of EO as well as the quantitative analyses of total possible bioactive compounds present in hydro-ethanolic and aqueous extracts of the plant (total phenolics, total flavonoids, and total proanthocyanidins). Up to date, no previous studies were reported on the metabolic profile of Egyptian *T. bovei* EO along with the above-mentioned pharmacologic properties. Therefore, in this study, we present the first report on such investigations in order to expand the medicinal value of this plant and hence promote its use in clinical use.

## 2. Experimental Section

### 2.1. Plant Materials

Aerial parts of *T. bovei* Benth. were collected from El-Hammam region (30.841852° N 29.394043° E), Egypt in Spring 2016. Plant materials have been authenticated by Dr. Sherif T.S. Hassan and voucher specimens (TBB1-2016) were deposited at the Herbarium of the Department of Natural Drugs, Faculty of Pharmacy, University of Veterinary and Pharmaceutical Sciences Brno, Czech Republic.

#### 2.1.1. EO Extraction

EO was obtained from air-dried aerial parts (410 g) by hydro-distillation method using Clevenger-type apparatus for 3 h following the recommendations of European Pharmacopeia. EO was gained with 1.54 % yield (*v/w*) [22].

#### 2.1.2. Phytochemical Profile Analysis of EO by GC and GC-MS

Gas chromatography (GC; Agilent 6890N, Santa Clara, CA, USA) equipped with a flame ionization detector (FID) and gas chromatography-mass spectrometry (GC-MS) utilizing GC (Agilent 5975, Santa Clara, CA, USA) linked with a mass selective detector (Hewlett Packard-MSD5973, Santa Clara, CA, USA) have been used for identification of EO components. Parameters such as the instrumental setup, operational, and chromatographic settings were tracked as described earlier [23]. EO constituents were identified by matching the obtained mass spectra with Mass Finder-4 Library and Wiley GC-MS Library and by comparison with data obtained from the literature [24,25,26].

### 2.2. Quantitative Analyses of Total Bioactive Constituents

#### 2.2.1. Extraction Procedure

The hydro-ethanolic extract of air-dried powder of *T. bovei* (HETB) was obtained by maceration for 3 days using ethanol:water (70:30 *v*/*v*) (1 g/10 mL), while the aqueous extract of air-dried powder *T. bovei* (AETB) (1 g/10 mL) was prepared by boiling in distilled water for 30 min and left to stand to cool down at 26 °C. The solutions were filtered by Sartorius 388 filter paper and the filtered extracts were employed for analyses.

##### Total Phenolic Content (TPC)

Folin-Ciocalteu method was used to determine the total phenolic contents (TPC) of HETB and AETB as described earlier [27]. TPC value was expressed as gallic acid equivalents (GAE) (mg GAE/g dry weight of plant material) (*R*^2^ = 0.99). Experiments were conducted in triplicate.

##### Total Flavonoid Content (TFC)

The total flavonoid content (TFC) of HETB and AETB was assessed and expressed as rutin equivalents following the method by Hassan et al. [28]. TFC value was expressed as rutin equivalents (RE) (mg RE/g dry weight of plant material) (*R*^2^ = 0.99). Experiments were assayed in triplicate.

##### Total Proanthocyanidin Content

The HCl/butan-1-ol assay was achieved to determine the total amount of proanthocyanidin in HETB and AETB and the total value was expressed as µg cyanidin chloride/g dry weight of plant material as previously described [29]. All measurements were analyzed in triplicates.

### 2.3. Cytotoxicity Profile against Human Carcinoma Cells

#### 2.3.1. Cell Lines, Medium and Reagents

Human cervical carcinoma cells (HeLa-R2), colon cancer cells (LS-174-D3), lung adenocarcinoma cells (A-549-C5), and normal human fetal lung fibroblast cells (MRC-5) were courtesy by Motol University Hospital (MUH), Prague, Czech Republic. The cells were cultivated as monolayer culture in a culture medium (Roswell Park Memorial Institute RPMI;1640; Sigma Chemicals Co., Saint Louis, MO, USA) supplied with streptomycin (200 mg/mL), penicillin (192 U/mL), L-glutamine (3 mM), 4-(2-hydroxyethyl) piperazine-1-ethanesulfonic acid (HEPES) (25 mM), and 10% of heat-inactivated fetal calf serum (FCS) at pH = 7.2. Further, the human cancer cells were grown in 5% CO_2_ and humidified condition at 37 °C, and subsequently sub-cultured twice for a week as previously described [30].

#### 2.3.2. Cytotoxicity Assay

The potential cytotoxic effect of TB essential oil (TB-EO) on various human carcinoma cells along with normal human fetal lung fibroblast cells was screened by MTT assay (3-(4,5-dimethylthiazol-2-yl)-2,5-diphenyltetrazolium Bromide) (Sigma-Aldrich, Berlin, Germany) as described earlier [30]. Cisplatin, a standard chemotherapy drug was selected as a reference control (Sigma-Aldrich, Prague, Czech Republic). The absorbance of the samples was detected at 570 nm using a microplate reader (Infinite M200, Tecan, Salzburg, Austria). Determination of the half-maximal inhibitory concentrations (IC_50_) was acquired from the cell survival diagrams that necessitate suppressing 50% of cell survival.

### 2.4. Antiviral Activity against HSV-2

#### 2.4.1. Viral Strains, Cell Lines, Medium, and Reagents

Vero cells (ATCC: CCL 81TM, UK; were gained from MUH, Prague, Czech Republic) were grown and prepared as previously described [31]. A clinical isolate of HSV-2 (obtained from HSV-2 infected patients) was kindly acquired from MUH, Prague, Czech Republic. The clinical strain was typed, identified, propagated in Vero cells, and the titers, which are expressed as 50% tissue culture infective dose (TCID_50_/mL), were assayed by cytopathic end-point method following the same procedures by Hassan et al. [31]. Viral stocks were stored at −80 °C.

#### 2.4.2. Determination of Cytotoxicity

**Figure 1:**
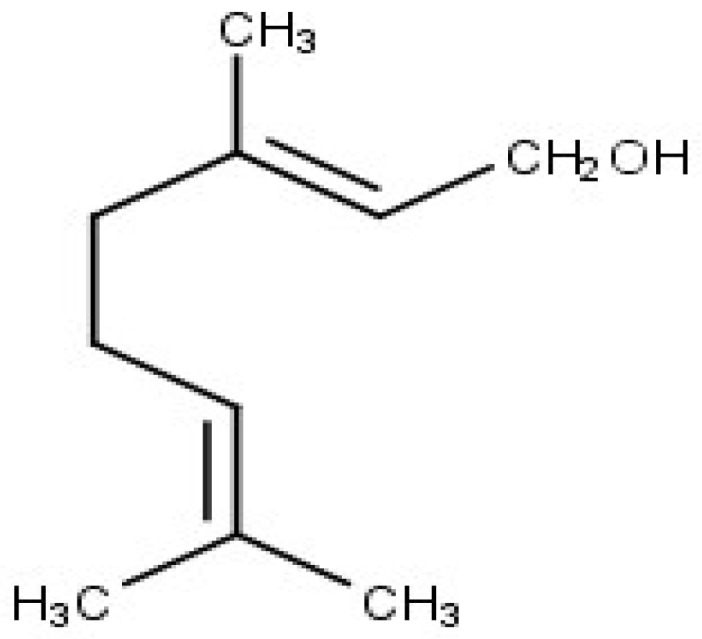
Chemical structure of geraniol.

The cytotoxicity was assayed by treating Vero cell monolayers with TB-EO, geraniol (Sigma-Aldrich, Prague, Czech Republic; Figure 1), and TB-EO with ACV (Sigma-Aldrich, Prague, Czech Republic) in combination, as well as standard ACV using the neutral red dye-uptake method as previously described [31]. The 50% cytotoxic concentrations (CC_50_) of test compounds were determined as the concentration that diminishes cell viability by 50% when compared to the untreated controls.

#### 2.4.3. Antiviral Activity Against HSV-2

Antiherpetic properties of TB-EO, geraniol, and TB-EO combined with ACV against HSV-2 replication were assayed by the titer reduction method as previously described [31]. ACV was used as a reference antiherpetic drug. The 50% effective concentration (EC_50_) was assessed as the concentration needed for 50% protection towards virus-induced cytopathic effects. Subsequently, a selectivity index (SI) was calculated as the ratio CC_50_/EC_50_.

#### 2.4.4. Molecular Docking Study of Geraniol into the Binding Site of HSV-2 Protease

##### Protein-Ligand Elaboration and Processing of Docking Analysis

The 3D-crystal structure of HSV-2 protease (PDB ID: 1AT3) and 3D-structure of geraniol (SDF file ID: 64Z) were acquired from the RCSB Protein Data Bank. PyRx docking tool within Autodock VINA software (The Scripps Research Institute, La Jolla, CA, USA) was used to evaluate the binding mode of geraniol in the active site of HSV-2 protease. All docking parameters, settings, calculations, protonation conditions, and the overall charges were tracked as previously described [32,33]. All docked complexes were presented graphically by Discovery studio visualizer version 4.0 (BIOVIA, San Diego, CA, USA) [34].

### 2.5. Anti-Angiotensin-Converting Enzyme (ACE) Activity

The catalytic activity of human ACE was assayed spectrophotometrically based on the formation of hippuric acid as a product following the method by Cushman and Cheung [35] with slight modification. Briefly, the activity was initiated by incubating 166 mU/mL of human ACE (Sigma-Aldrich, Prague, Czech Republic) prepared in Tris Buffer (50 mM, pH = 8.3) with TB-EO, geraniol, and captopril (standard inhibitor) at concentrations of 15 µg/mL for 80 min at 37 °C. Further, the catalytic reaction took place by adding the mixture to 110 μL of the 10 mM hippuryl-histidyl-leucine (substrate; from Sigma-Aldrich, Prague, Czech Republic). The absorbance of the hippuric acid was detected at λ 228 nm, and the % inhibition was calculated.

### 2.6. Molecular Docking Study of Geraniol into the Binding Site of ACE

#### Protein-Ligand Preparation and Processing of Docking Analysis

The 3D-crystal structure of human angiotensin-converting enzyme (ACE) complexed with captopril (PDB ID: 1UZF) and 3D-structure of geraniol (SDF file ID: 64Z) were attained by the RCSB Protein Data Bank. Before performing the docking analysis, we authenticated the docking process by removing the co-crystallized ligand captopril from the PDB (PDB ID: 1UZF) structure and re-docked captopril using PyRx docking tool via Autodock VINA software. This software was used to evaluate the binding mode of geraniol in the active site of human ACE. All docking settings, parameters, calculations, protonation conditions, and the overall charges were followed as previously described [32]. All docked complexes were presented graphically using Discovery studio visualizer version 4.0 (BIOVIA, San Diego, CA, USA) [34].

## 3. Results and Discussion

### 3.1. Characterization of EO

The metabolic profile of TB-EO (99.9%) is characterized by geraniol (32.3%), α-citral (27.7%), β-citral (12.4%), thymol (3.8%), dl-camphor (2.4%), 1,8-cineole (2.3%), 3-octanol (2.2%), camphene (1.8%), farnesene (1.7%), isocaryophyllene (1.6%), β-ocimene (1.5%), α-limonene (1.5%), β-myrcene (1.4%), α-phellandrene (1.4%), β-linalool (1.3%), β-pinene (1.3%), α-terpinene (1.2%), and nerolidol (1.1%) as the principal components (Table 1). Interestingly, the oxygenated monoterpenes appeared to have the highest percentage (82.2%), where geraniol (32.3%), α-citral (27.7%), and β-citral (12.4%) have formed the highest percentage of oxygenated monoterpenes. Monoterpene hydrocarbons were found to be (10.1%), while sesquiterpene hydrocarbons were accounted to be (3.3%). Moreover, oxygenated sesquiterpenes (1.1%) and the non-terpenoid compound 3-octanol (2.2%) have been identified. It is worth to declare that our results are in total agreement with those results that have reported geraniol, α-citral, and β-citral as the primary constituents of EO of *T. bovei* [3]. On the other hand, some investigations have presented carvacrol, thymol, and *p*-cymene as the main metabolites detected in Turkish *T. bovei* EO [5,36,37]. These differences in the chemical composition could be correlated to several aspects including but not limited to the isolation procedures used, the impact of genetic, environmental, climate, and growing conditions, and in addition to the impact of organ age [23,38,39].

### 3.2. Characterization of Total Bioactive Compounds

The quantitative analyses of total bioactive constituents (TPC, TFC, and total proanthocyanidin content) of HETB and AETB were determined spectrophotometrically. The results are shown in Table 2. The contents of all studied phenolic constituents of HETB were observed to be higher than that of AETB. These results suggest the significance of used solvents. The polyphenols are more soluble in ethanol, therefore, HETB gave higher yields than AETB. Our findings agree with the literature, where methanol or ethanol extracts were reported to show the highest levels of total phenolics, flavonoids, and proanthocyanidins [40,41]. However, several studies describe the water as the most appropriate solvent for the acquisition of the highest phenolic contents from the plant material [42,43]. Our results indicate that *T. bovei* contains a lower amount of phenolic compounds extracted by ethanol with the value of TPC 85.62 mg/g DW of plant material in comparison with other ethanol extracts of *Thymus* species. *T. vulgaris* L. reached the value of TPC 158.0 µg/mg [43], *T. serpyllum* 113.0 mg/g [44] and *T. pulegioides* L. 155.38 mg/g of HEE [45]. On the other hand, TFC value of *T. bovei* (51.23 ± 0.43 mg/g) was found to be higher than that of different *Thymus* species by using an ethanol for extraction and the same procedure to determine the TFC values. Nickavar and Esbati [46] have reported the TFC value 50.39 µg/mg for *T. pubescens* Boiss. & Kotschy ex Celak., 37.11 µg/mg for *T. kotschyanus* Boiss. & Hohen., and 35.21 µg/mg for *T. daenensis* Celak., respectively. The TPC and TFC values may significantly vary during the season as it was observed in *T. longicaulis* [47]. The habitat also plays an important role in the total content of the polyphenolics. Jaouadi et al. [48] have described the variation of phenolic constituents of *T. capitatus* (L.) Hoffmanns. & Link harvested in six bioclimatic zones and Tohidi et al. [49] have demonstrated the variations in chemical constituents of fourteen *Thymus* accessions collected from different regions of Iran.

### 3.3. Evaluation of Cytotoxicity Properties

Over the past decade, cancer has shown the capacity to progress resistance to traditional medications, and the growing frequency of drug-resistant cancer imposes an urgent need to operate further research and treatment development. In this study, we assessed the cytotoxicity profile of TB-EO in comparison with the reference chemotherapeutic drug cisplatin towards various human cancer cell lines, along with normal human fetal lung fibroblast cells. The results exposed that TB-EO exerted remarked cytotoxic properties against human cervical carcinoma cells (HeLa-R2), colon cancer cells (LS-174-D3), and lung adenocarcinoma cells (A-549-C5) with IC_50_ values of 7.22, 9.30, and 8.62 µg/mL, respectively, in comparison with that of cisplatin with IC_50_ values of 4.24, 5.21, and 5.43 µg/mL, respectively (Table 3). Among the obtained results, TB-EO showed strong cytotoxicity against HeLa-R2 cells with an IC_50_ value of 7.22 µg/mL. Interestingly, TB-EO showed very weak cytotoxicity on the normal human fetal lung fibroblast cells (MRC-5) with an IC_50_ value of 118.34 µg/mL compared with that of cisplatin (IC_50_ = 10.08 µg/mL). This indicates that TB-EO has powerful anti-tumor properties on cancer cells and no significant effect on normal cells. The cytotoxic effect of TB-EO used in this study could be attributed to the major compounds (geraniol, α-citral, β-citral, and thymol) present in TB-EO. In this study, we present the first report on the cytotoxic activities of Egyptian TB-EO. However, several studies have explored variable data of toxicology profiles of EOs on cancer cells from different *Thymus* species, except *T. bovei* [50,51,52], we cannot compare our results with them due to the variation in test methods used, as well as different cell lines sources and samples used. Finally, our results suggest that TB-EO might be used to treat cancer without affecting normal cells.

### 3.4. Antiviral Activities against HSV-2 Replication

Prior to carrying out the anti-HSV-2 assay, we evaluated the cytotoxicity of TB-EO, geraniol, TB-EO combined with ACV as well as standard ACV in Vero cells by the neutral red dye-uptake technique. The selectivity index is vital to control the probable toxic property of any medication on the cells that could be confused with an antiviral activity. The CC_50_ values of test compounds were determined to be greater than 210 µg/mL (Table 4). Antiherpetic activity was assayed in infected Vero cells, by titer reduction assay using quantitative real-time reverse transcription PCR. TB-EO, geraniol, TB-EO combined with ACV along with standard ACV, have displayed pronounced inhibitory properties against the replication of HSV-2 with EC_50_ values of 2.13, 1.92, 0.81 and 1.94 µg/mL, respectively with corresponding selectivity indices 98.59, 109.38, 259.26 and 108.25 (Table 4). Our findings declare that geraniol (an active constituent in TB-EO) possesses similar anti-HSV-2 activity (EC_50_ = 1.92 µg/mL; SI: 109.38) compared with that of ACV (EC_50_ = 1.94 µg/mL; SI: 108.25). Interestingly, TB-EO with ACV in combination exhibited a great synergy effect, which in turn enhanced the potency of antiherpetic activity against HSV-2 replication (EC_50_ = 0.81 µg/mL; SI: 259.26) compared with that of ACV (EC_50_ = 1.94 µg/mL; SI: 108.25). On the other hand, TB-EO exerted lower anti-HSV-2 activity (EC_50_ = 2.13 µg/mL; SI: 98.59) than ACV (EC_50_ = 1.94 µg/mL; SI: 108.25).

In recent years, natural products have attracted the attention of the pharmaceutical industry as an alternative or complementary therapy to the currently existing ones for the treatment of viral infections [19]. The current pharmacologic treatment of HSV infections is based on the use of ACV and related synthetic nucleoside analogs, which interact with viral DNA replication via activation by viral thymidine kinase [33]. The increasing use of antiviral drugs to treat HSV infections has resulted in drug resistance problem, which in return has switched the attention to plant-derived products in clinical practice [53]. Generally, antiviral medications are categorized into three groups: virucidals, immunomodulators, and antiviral chemotherapeutic drugs [19]. Based on the obtained results, we might classify the test compounds as antiviral chemotherapeutic agents. It has been stated that the resistance to antiviral drugs can be overcome by merging them with compounds from different classes of origin [54,55]. Thus, the results gained by combining TB-EO with ACV have led to potent anti-HSV-2 properties along with less resistance. Remarkably, TB-EO, geraniol, and TB-EO combined with ACV seem to be anti-HSV-2 drugs of future that could be used for the treatment of HSV-2 infection.

### 3.5. Molecular Interactions of Geraniol with HSV-2 Protease

**Figure 2:**
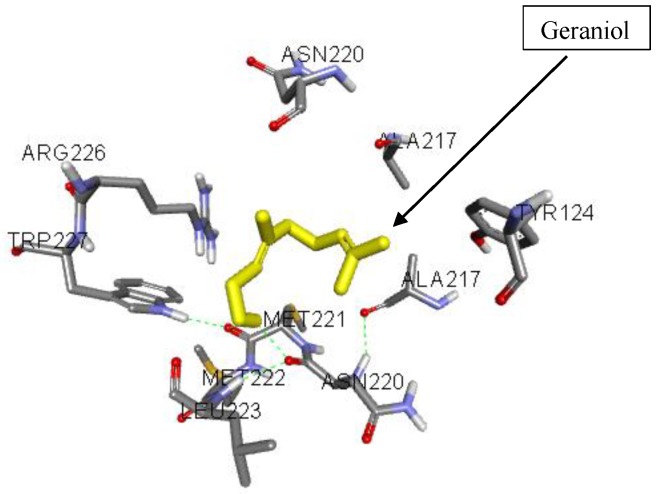
3D interaction diagram of geraniol in the active cavity of HSV-2 protease.

**Figure 3:**
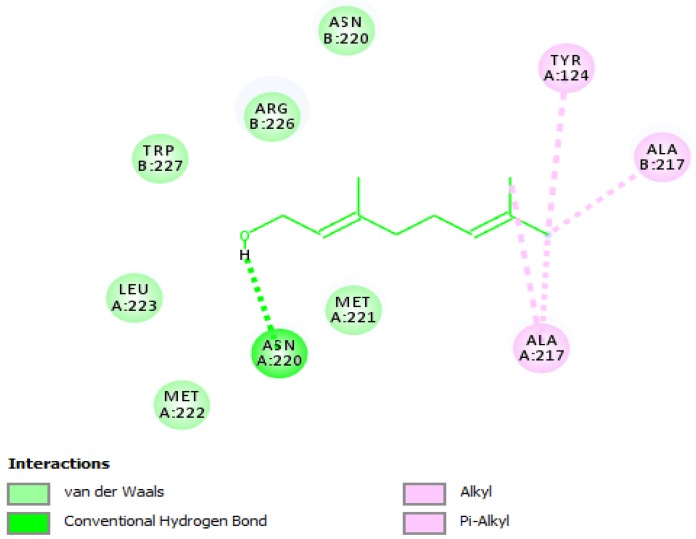
2D interaction diagram of geraniol in the active cavity of HSV-2 protease. Only those amino acid residues implicated in the enzyme stabilization are exposed. Hydrogen bonding and several substantial interactions with amino acid residues are displayed.

It is known that HSV-2 encodes a serine protease that is vital for viral replication and hence represents a viable target for therapeutic intervention. Thus, a molecular docking study was carried out to unveil the binding mode of geraniol into the active site of HSV-2 protease (Figure 2). The docking score for geraniol, which is expressed as binding affinity, was recorded to be −5.4 kcal/mol. The docking results showed that geraniol bound to the active site of HSV-2 protease and hence inhibited the enzyme by establishing a hydrogen bonding contact with the hydroxyl group of geraniol and amino acid residues ASN-A220. Additional hydrophobic and other critical interactions were recognized (Figure 3). As shown in Figure 3, all amino acid residues were previously reported in the active site of HSV-2 protease and are responsible for the stabilization of the enzyme [56]. Since geraniol bound to the active-site of the enzyme, this means that geraniol is competing with the substrate, and hence acts as a competitive inhibitor.

### 3.6. Evaluation of Anti-ACE Properties

Inhibition of ACE is one of the most imperative strategies to control hypertension and heart failure. The degree of ACE inactivation by TB-EO, geraniol, and captopril was signified by the amount of hippuric acid formed. Based on the estimated percentage of inhibition, TB-EO (15 µg/mL), and geraniol (15 µg/mL) showed prominent inhibitory activities against ACE with % inhibition 95.4 and 92.2, respectively, compared to captopril (99.8 % at a concentration of 15 µg/mL) (Table 5). Since TB-EO inactivated potently the hydrolytic activities of ACE, we decided to test the principal active compound in TB-EO, geraniol for further evaluation against ACE. Based on the obtained results, geraniol remarkably suppressed the hydrolytic properties of ACE. Accordingly, we may suggest that the inhibitory effect of TB-EO on ACE is attributed to the presence of geraniol. In this study, we present a first report on the inhibitory activities of TB-EO and its bioactive molecule geraniol against ACE. Despite limited investigations have confirmed that plant EOs have processed in vitro anti-ACE activities, we tried to rationalize our results with such studies. For instance, Zouari et al. [57] stated that *Artemisia herba-alba* Asso. EO unveiled in vitro ACE inhibitory action at a concentration of 100 µg/mL (25.4%). In another study, *Ajuga pseudoiva* Rob. EO was pronounced to exert a dose-dependent ACE inhibitory activity of 28.3%, 53.8%, and 74.5%, at 25, 75 and 150 µg/mL of EO, respectively [58]. On the other hand, compounds derived from plant origins such as flavonoids, tannins, and peptides were described with anti-ACE activities [59,60,61].

### 3.7. Molecular Interactions of Geraniol with ACE

**Figure 4:**
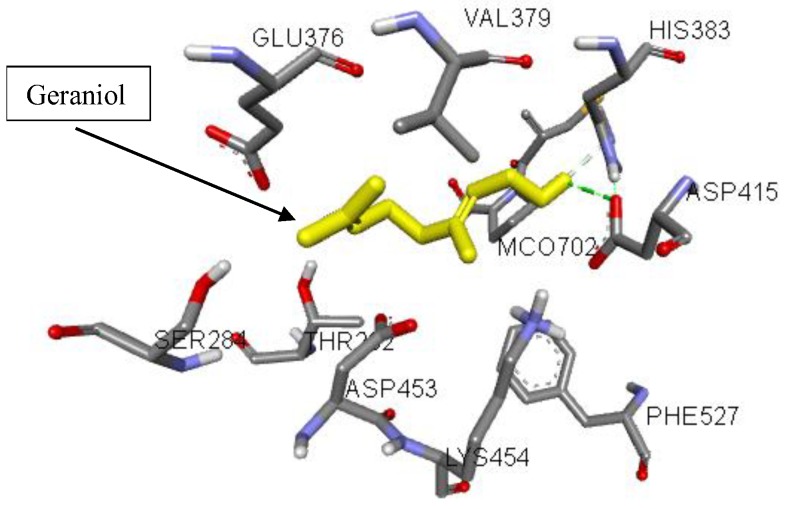
3D interaction diagram of geraniol in the active cavity of the human ACE.

**Figure 5:**
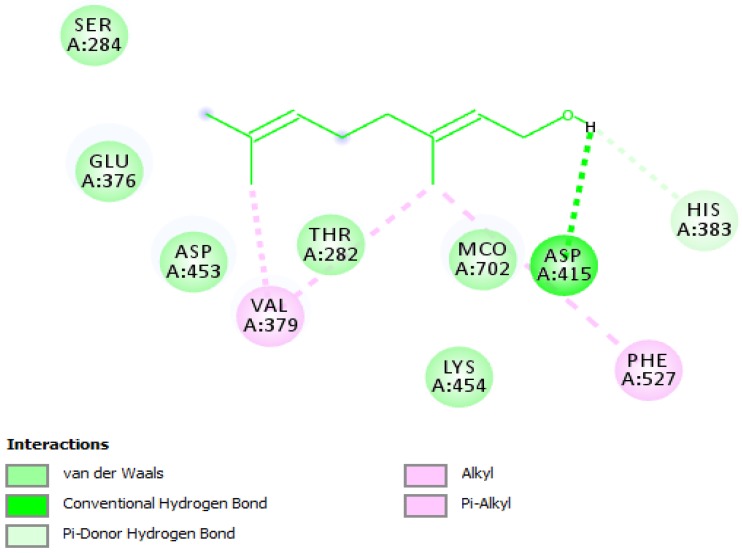
2D diagram shows the binding mode of geraniol in the active cavity of the ACE. Only those amino acid residues embroiled in ACE stabilization are viewed. Hydrogen bonding and several fundamental interactions with corresponding amino acid residues are presented.

To better understand the potency of geraniol for further structure-activity relationship study, molecular docking analysis with ACE was conducted to predict the binding affinity of geraniol into the binding site of ACE (Figure 4), which contributes to rationalize the obtained biological results as well as the mechanism of action. The docking scores for geraniol and captopril, which are expressed as binding affinities, were found to be −5.2 and −6.1 kcal/mol, respectively. The docking results revealed that geraniol pound to the active cavity of ACE by forming a hydrogen bonding interaction between amino acid residue ASP-415 and the hydroxyl group of geraniol, which in turn resulted in suppressing enzymatic activity (Figure 5). Moreover, several critical interactions were also observed. As shown in Figure 5, all amino acid residues were previously described in the active cavity of human ACE and found to be critical for the stabilization of the enzyme [62]. Although zinc is essential to the catalytic activity of ACE, geraniol did not bind to the zinc active-site and bound to the different active pocket of the enzyme. This means that geraniol is not competing with the substrate, and hence acts as a non-competitive inhibitor by preventing the formation of enzyme-product complexes.

## 4. Conclusions

Recently, plant-derived EOs have attracted the attention of healthcare providers worldwide due to the therapeutic properties against various diseases as well as the safety profiles. Our study showed that *T. bovei* EO exerted remarked cytotoxic properties against human cervical carcinoma cells, colon cancer cells, and lung adenocarcinoma cells without affecting normal cells compared with that of cisplatin based on the determination of IC_50_ concentrations that required to inhibit 50% of cell survival (see results and discussion section). Additionally, geraniol, the main component of EO, showed prominent inhibition activities against HSV-2 replication and suppressing the activity of ACE (see results and discussion section). Hereby, we present geraniol as a very promising agent in the treatment of genital herpes and hypertension. Subsequently, we confirmed the mechanism of action by using molecular docking studies. These studies have confirmed the basics of phytotherapeutical use of *T. bovei* in Mediterranean region and its inclusion in North African pharmacopoeias. Finally, further studies should be conducted in vivo and in clinical trials to evaluate efficacy as well as pharmacokinetic and pharmacodynamic properties, leading to effective optimization of TB-EO and geraniol as natural cytotoxic, antiherpetic, and antihypertensive agents. Moreover, large prospective epidemiological studies should be performed.

## Figures and Tables

**Table 1:** The metabolic profile of TB-EO.

Identified Compounds	*T. Bovei* Benth. (%) ^a^	RRI ^b^	Identification Process
α-citral (*Trans*-citral)	27.7	1140	t_R_, MS
β-citral (*Cis*-citral, neral)	12.4	1194	t_R_, MS
Geraniol (Lemonol)	32.3	1209	t_R_, MS
dl-camphor	2.4	1430	t_R_, MS
3-octanol	2.2	1461	t_R_, MS
1,8-cineole (Eucalyptol)	2.3	1531	t_R_, MS
Thymol	3.8	1578	t_R_, MS
*p*-Cymene	0.1	1593	t_R_, MS
Geranyl isobutyrate	0.1	1681	t_R_, MS
β-linalool	1.3	1764	t_R_, MS
Geranyl propionate	0.2	1915	t_R_, MS
α-cyclocitral	0.2	2028	t_R_, MS
Linalyl acetate (Bergamol)	0.2	2071	t_R_, MS
Camphene	1.8	2075	t_R_, MS
Isocaryophyllene	1.6	2107	t_R_, MS
Farnesene	1.7	2212	t_R_, MS
Dihydrocarveol acetate	0.1	2404	t_R_, MS
β-myrcene	1.4	2621	t_R_, MS
l-borneol	0.1	2801	t_R_, MS
β-ocimene	1.5	2839	t_R_, MS
Nerolidol	1.1	2873	t_R_, MS
α-limonene	1.5	2903	t_R_, MS
α-terpinene	1.2	2944	t_R_, MS
α-phellandrene	1.4	2976	t_R_, MS
β-pinene	1.3	2995	t_R_, MS
Total	99.9		
Oxygenated monoterpenes	82.2		
Monoterpene hydrocarbons	10.1		
Sesquiterpene hydrocarbons	3.3		
Oxygenated sesquiterpenes	1.1		
Others	3.2		

Percentage (%) ^a^ assessed from flame ionization detector (FID) data for the polar column; RRI ^b^: Relative retention indices calculated against *n*-alkanes on the polar column; tr, trace (<0.1%); t_R_: Retention times of genuine compounds on the HP Innowax column; MS: Mass spectrometry—the identification is based on the comparison of the mass spectra with those of the Wiley and Mass Finder libraries as well as literature data.

**Table 2:** Chemical characterization of TPC and TFC and total proanthocyanidins in HETB and AETB.

Extract	TPC (mg GAE/g DW of Plant Mterial)	TFC (mg RE/g DW of Plant Material)	Total Proanthocyanidins (µg CC/g DW of Plant Material)
HETB	85.62 ± 0.82	51.23 ± 0.43	135.91 ± 0.83
AETB	74.84 ± 1.43	40.51 ± 0.64	110.31 ± 1.12

Values are expressed as the mean ± standard deviation (SD). Experiments were assayed in triplicates. TPC: Total phenolic content; TFC: Total flavonoid content; HETB: Hydro-ethanolic extract of *T. bovei*; AETB: Aqueous extract of *T. bovei*; GAE: Gallic acid; RE: Rutin; CC: Cyanidin chloride; DW: Dry weight.

**Table 3:** Cytotoxic effect of TB-EO on human cancer cell lines.

	IC_50_ (µg/mL)			
Compound	HeLa-R2	LS-174-D3	A-549-C5	>MRC-5
TB-EO	7.22 ± 1.21	9.30 ± 0.84	8.62 ± 1.41	118.34 ± 0.56
Cisplatin	4.24 ± 0.81	5.21 ± 0.23	5.43 ± 0.34	10.08 ± 0.71

Values presented are means ± standard deviation (SD) of three independent experiments performed in triplicates. HeLa-R2: Human cervical carcinoma cells; LS-174-D3: Colon cancer cells; A-549-C5: Lung adenocarcinoma cells; MRC-5: Normal human fetal lung fibroblast cells; TB-EO: *T. bovei* essential oil; IC_50_: The concentration of drug that possesses 50% inhibition of cell survival.

**Table 4:** Antiviral activity of TB-EO, geraniol, and TB-EO with ACV in combination against HSV-2 replication.

Test Compounds	CC_50_ (µg/mL)	EC_50_ (µg/mL)	SI
TB-EO	>210	2.13 ± 0.63	>98.59
Geraniol	>210	1.92 ± 0.84	>109.38
TB-EO combined with ACV	>210	0.81 ± 1.21	>259.26
ACV	>210	1.94 ± 0.41	>108.25

Values demonstrated are means ± standard deviation (SD) of three independent experiments conducted in duplicate. TB-EO: *T. bovei* essential oil; ACV: Acyclovir; CC_50_: 50% cytotoxic concentration; EC_50_: 50% effective concentration; SI: Selectivity index (calculated as the ratio CC_50_/EC_50_). Experiments were performed in duplicate in three independent experiments. PRISM software version 5.0 (GraphPad Software, Inc., La Jolla, CA, USA) was used for statistical analysis (one-way ANOVA test) and to calculate EC_50_ and CC_50_ parameters.

**Table 5:** In vitro ACE inhibitory activities of TB-EO, geraniol, and captopril.

Compounds	% Inhibition
TB-EO	95.4 ± 0.94
Geraniol	92.2 ± 1.14
Captopril	99.8 ± 1.21
Catalyzed reaction (no inhibition)	Nd

Values are presented as the mean ± standard deviation (SD) (*n* = 3). TB-EO: *T. bovei* essential oil; Nd: Not determined.
